# The Impact of Water Intrusion on Pathogenic *Vibrio* Species to Inland Brackish Waters of China

**DOI:** 10.3390/ijerph17186781

**Published:** 2020-09-17

**Authors:** Qingyao Wang, Songzhe Fu, Qian Yang, Jingwei Hao, Can Zhou, Ying Liu

**Affiliations:** 1 College of Marine Technology and Environment, Dalian Ocean University, Dalian 116023, China; wangqingyao0227@163.com (Q.W.); haojingw@foxmail.com (J.H.); zhoucan1230@163.com (C.Z.); yingliu@dlou.edu.cn (Y.L.); 2 Key Laboratory of Environment Controlled Aquaculture (KLECA), Ministry of Education, Dalian 116023, China; 3 Center for Microbial Ecology and Technology (CMET), Ghent University, Coupure Links 653, 9000 Gent, Belgium

**Keywords:** seawater intrusion, *Vibrio parahaemolyticus*, *Vibrio cholerae*, MLST, virulence factor

## Abstract

The estuary is the ecological niche of pathogenic *Vibrio* spp. as it provides abundant organic and inorganic nutrients from seawater and rivers. However, little is known about the ecology of these *Vibrio* species in the inland brackish water area. In this study, their co-occurrence and relationships to key environmental constraints (salinity and temperature) in the Hun-Tai River of China were examined using the most probable number polymerase chain reaction (MPN-PCR) approach. We hereby report 2-year continuous surveillance based on six water indices of the Hun-Tai River. The results showed that seawater intrusion maximally reached inland as far as 26.5 km for the Hun-Tai River. Pathogenic *Vibrio* spp. were detected in 21.9% of the water samples. In particular, *V. cholerae*, *V. parahaemolyticus*, and *V. vulnificus* were isolated in 10 (10.4%), 20 (20.8.5%), and 2 (2.08%) samples, respectively. All *V. parahaemolyticus* strains were *tdh* gene negative, 10% were positive for the *trh* gene. Multi-locus sequence typing (MLST) divided *V. parahaemolyticus* strains into 12 sequence types (STs) for the Hun-Tai River. Five STs were respectively present in various locations along the Hun-Tai River. The PCR assay for detecting six virulence genes and *Vibrio* seventh pandemic island I and II revealed three genotypes in 12 *V. cholerae* isolates. The results of our study showed that seawater intrusion and salinity have profound effects on the distribution of pathogenic *Vibrio* spp. in the inland river, suggesting a potential health risk associated with the waters of the Hun-Tai River used for irrigation and drinking.

## 1. Introduction

*Vibrio* spp. are a group of Gram-negative, rod-shaped bacterial species that are a natural constituent of freshwater, estuarine, and marine environments [1]. Numerous studies show that the estuary is the ecological niche of *Vibrio* spp. as it provides abundant organic and inorganic nutrients both from seawater and rivers. The species of significant clinical importance include *V*. *cholerae*, *V*. *parahaemolyticus*, and *V*. *vulnificus* [2]. *V*. *cholerae* is the causal agent of cholera, which is an acute life-threatening diarrheal disease occurring in many developing countries [3]. *V. parahaemolyticus* causes the symptoms of acute gastroenteritis, which is often associated with the consumption of raw or undercooked seafood [4]. *V*. *vulnificus* can cause wound infections and septicemia in immunocompromised individuals or individuals with liver disease [5].

From a One Health perspective, the inland movement of the salt–freshwater transition zone would potentially introduce pathogenic *Vibrio* spp. into inland water, resulting in community outbreaks. However, the majority of studies have focused on the distribution and prevalence of pathogenic *Vibrio* spp. in the estuary [6,7,8]. Few studies have investigated the dissemination of pathogenic *Vibrio* spp. from the estuary to the inland river [9]. Several molecular fingerprinting methods, such as pulsed-field gel electrophoresis (PFGE), denaturing gradient gel electrophoresis (DGGE), and Errterobacterial Repetitive Intergenic Consensus polymerase chain reaction (ERIC-PCR) have been adopted to subtype the pathogenic *Vibrio* spp. [10,11]. However, the relatively low resolution of these techniques may result in an incomplete or even biased estimation of the genetic diversity of *Vibrio* spp. More importantly, the fingerprinting results cannot be shared globally due to the use of different sets of restriction enzymes. Multi-locus sequence typing (MLST), on the other hand, is an unambiguous procedure for characterizing isolates of bacterial species using the sequences of seven housekeeping genes, which can be shared and standardized globally [12]. The MLST scheme for *Vibrio* spp. has been recently established to identify the genotypes for *Vibrio* spp. [13]. This state-of-the-art technique represents a promising tool for providing a low-cost unified tool for typing pathogenic *Vibrio* spp., which would provide valuable information for understanding the distribution of the distinct genotype in rivers.

**Figure 1:**
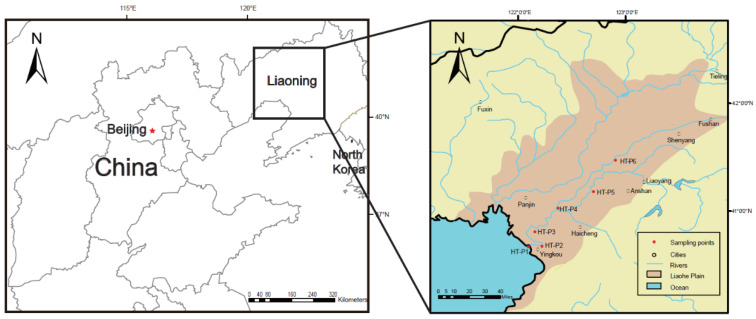
Sampling sites in this study. The sampling positions on the Hun-Tai River are indicated in the square. The sampling sites were mapped by ArcGIS Desktop 10.2 software (http://desktop.arcgis.com/). Sampling sites on the Hun-Tai (HT) River include estuary wetland (HT-P1), Dukou Port (HT-P2), Shuiyuan (HT-P3), Shifou (HT-P4), Liaoyang (HT-P5), and Tongerpu (HT-P6).

The Hun-Tai (HT) River, located in Liaoning Province, China, is the sole source of potable water for the majority of cities along the HT River (Figure 1). Consequently, pathogen surveillance along the river sites used for drinking is critical for public health as it is severely impacted by seawater intrusion. The estuary of the HT River was an important port in north China, which was the hotspot of *V. cholerae* historically. In 1946 alone, there were 11 cholera outbreaks in this region, with more than 2600 deaths [3]. In our previous study, we conducted a six-month pathogen surveillance of this region and isolated non-O1/O139 *V*. *cholerae* in three sites of the HT River, which was linked to a human infection case [14].

To assess the health risk downstream of the HT River, in this study, we selected six sites in the river to investigate the inland distribution of three pathogenic *Vibrio* spp. systematically. The correlations between the key environmental variables and three pathogenic *Vibrio* spp. were monitored to assess the impacts of pathogenic *Vibrio* spp. strains on the drinking water sources in the HT River.

## 2. Materials and Methods

### 2.1. Study Area and Sample Collection

The Hun-Tai River, located in northeast China, belongs to the Liaohe River system and consists of the Hun River (415 km in length) and the Taizi River (413 km in length) (Figure 1). The Hun-Tai River drains across Liaohe Plain, which is one of the most densely populated plain areas and is an essential heavy industry region in China. The first study area is the lowland part of the Liaohe Plain (Figure 1), which covered the central metropolitan area of Liaoning Province, including the cities of Shenyang, Anshan, Fushun, Benxi, Liaoyang, Qingyuan, and Xinbin and covered an area of approximately 52,000 km^2^. The water in this region is heavily used by municipal and agricultural activities, which has also been subject to significant contamination from heavy metals, organics, and a range of other pollutants [15].

Six sites were selected for study along the HT River, China. The selection of the sampling sites was based on the distinct distribution of the salinity. The sites are situated from the estuary to the inland place with a distance of 95 km to the seashore, including estuary (HT-P1), Dukou (HT-P2), Shuiyuan (HT-P3), Shifou (HT-P4), Liaoyang (HT-P5), and Tongerpu (HT-P6). Pristine groundwater samples used for irrigation and fish farming inland and at the estuary were sampled (Figure 1). Water temperature was measured at 20 cm below the surface. Site descriptions and salinity data are summarized in Table 1. Average salinity was 24.3 ppt at HT-P1 (coastal area), 7.5 ppt at HT-P2 (mouth of river), 3.02 ppt at HT-P3 (brackish water area), and 0.66 ppt at HT-P4 (brackish water area), while HT-P5 and HT-P6 were freshwater zones. Three-liter samples of water were collected from the surface of the river near the water intakes of the drinking-water plant in four sterile 1 L polycarbonate bottles. For HT-P5, water samples were obtained from water pumped from the bottom of a fish farm. The water temperature was measured using a glass thermometer and salinity was measured in the laboratory using an electrical conductivity meter.

From 2018 to 2019, water samples were collected from the HT River. Monthly sampling was conducted from April to November, which covered spring (April to June), summer (July to August), and autumn (September to November). Sampling was not conducted during the remaining months since some of the water sampling sites were frozen. All samples were collected in triplicate at each site and were immediately transported within 8 h to the laboratory for analysis.

### 2.2. Abundance Analysis of V. cholerae, V. parahaemolyticus, and V. vulnificus

The presence of three pathogenic *Vibrio* spp. in water samples was investigated using the most probable number polymerase chain reaction (MPN-PCR) method according to the modified ISO 8914 standard method (ISO 8914:1990) [16]. Briefly, 500 mL of each water sample were concentrated by filtration through a 0.45 μm pore filter (Sartorius, Goettingen, Germany) with a vacuum pump, after which the filters were placed in 225 mL of alkaline peptone water (APW; 1% (wt/vol) peptone, 1% (wt/vol) sodium chloride; pH 8.2). After incubation at 30 °C for 18 h, 1 mL aliquots of enrichment broths showing growth were analyzed by PCR to detect the presence of a species-specific *toxR* gene for *V. parahaemolyticus* and *V. cholerae* [17] and the *vvhA* gene for *V. vulnificus* [18].

Moreover, to obtain the isolates, 0.1 mL aliquots of enrichment broths were plated on thiosulfate–citrate–bile salts–sucrose (TCBS) (Oxoid, Hampshire, UK) and CHROMagar Vibrio (CHROMagar Microbiology, Paris, France) agar plates and were incubated for 18 h at 30 °C. Presumptive *Vibrio* spp. colonies were subcultured on TSA. A partial sequence of the 16S rRNA of *Vibrio* species was amplified by PCR and submitted for sequencing for bacterial identification [19]. Bacterial isolates were stored at −80 °C in LB broth containing 50% (*v*/*v*) glycerol.

### 2.3. MLST Typing of V. parahaemolyticus and V. cholerae

Seven housekeeping genes were chosen as target genes for MLST analysis of *V. parahaemolyticus* (*adk*, *metE*, *mdh*, *pntA*, *pyrC*, *gyrB*, *purM*) and *V. cholerae* (*dnaE*, *dtdS*, *pntA*, *recA*, *gyrB*, *pyrC*, *tnaA*), while a 10-gene MLST scheme was used for *V. vulnificus* [12,20,21]. The primers and PCR amplification protocol are described on the pubMLST website (http://www.pubmlst.org). The amplicons were analyzed by agarose gel electrophoresis and photographed in a gel imaging system. Sequencing was performed at Beijing BGI-GBI Biotech Co., Ltd. The nucleotide sequences and deduced protein sequences were analyzed with EditSeq and Megalign software (DNASTAR, Madison, WI, USA).

### 2.4. Detection of Virulence Genes

The virulence genes of *V. parahaemolyticus*, including *tdh*, *trh*, and *tlh*, were tested by PCR amplification [22,23,24] (Table S1). For *V. cholerae*, the PCR for the genes *hlyA* (encoding hemolysin), *zot* (encoding zonula occludens toxin), *ctxA* (encoding cholera toxin), *tcpA* (encoding toxin co-regulated pilus), and *rtxA* and *rtxC* (encoding repeats in the structural toxin (RTX)) was carried out as described previously in a final volume of 20 µL [17,25,26,27,28]. In addition, PCR was used to screen for five genes in the *Vibrio* seventh pandemic island I (VSP-I) cluster (VC0175, VC0178, VC0180, VC0183, and VC0185) and eight genes in the VSP-II cluster (VC0490, VC0493, VC0498, VC0502, VC0504, VC0512, VC0514, and VC0516) [29]. Four virulence genes, *vvhA*, *viuB*, *rtxA*, and *pilA*, were selected to assess the pathogenicity of *V. vulnificus* [18].

### 2.5. Statistical Analysis

All statistical tests were considered significant at *p* < 0.05. Data of environmental parameters are shown as mean ± standard deviation. Spatial and temporal statistically significant differences among samples were evaluated through Mann–Whitney rank sum tests analysis of variance. Spearman correlations (rs) was used to detect the correlations between the presence of *V. vulnificus* and *V. parahaemolyticus* and water temperature and salinity. All statistical analysis was performed in SPSS version 20.0 (SPSS, Chicago, IL, USA).

## 3. Results

### 3.1. Characterization of Seawater Intrusion in the HT River

As shown in Table 1, the seasonal fluctuation of salinity in the HT River was monitored. In this study, a salinity value below 0.5 ppt was defined as fresh water. Results showed that seawater intrusion maximally reached inland as far as 26.5 km (HT-P4) for the HT River in spring (dry season). In the normal season (autumn), the freshwater zone becomes brackish at HT-P4 while the transition between the freshwater and the saltwater zones begins at HT-P3 (around 20.5 km to the seashore) in the rainy season (summer). There was no significant difference in water temperature among the five sites. Overall, salinity was lowest in summer since the majority of the rainfall occurs during the summer in north China.

### 3.2. The Abundance of V. cholerae, V. parahaemolyticus, and V. vulnificus

The abundance of three pathogenic *Vibrio* spp. and their relationship with water temperature and salinity from five sampling sites were determined.

**Figure 2:**
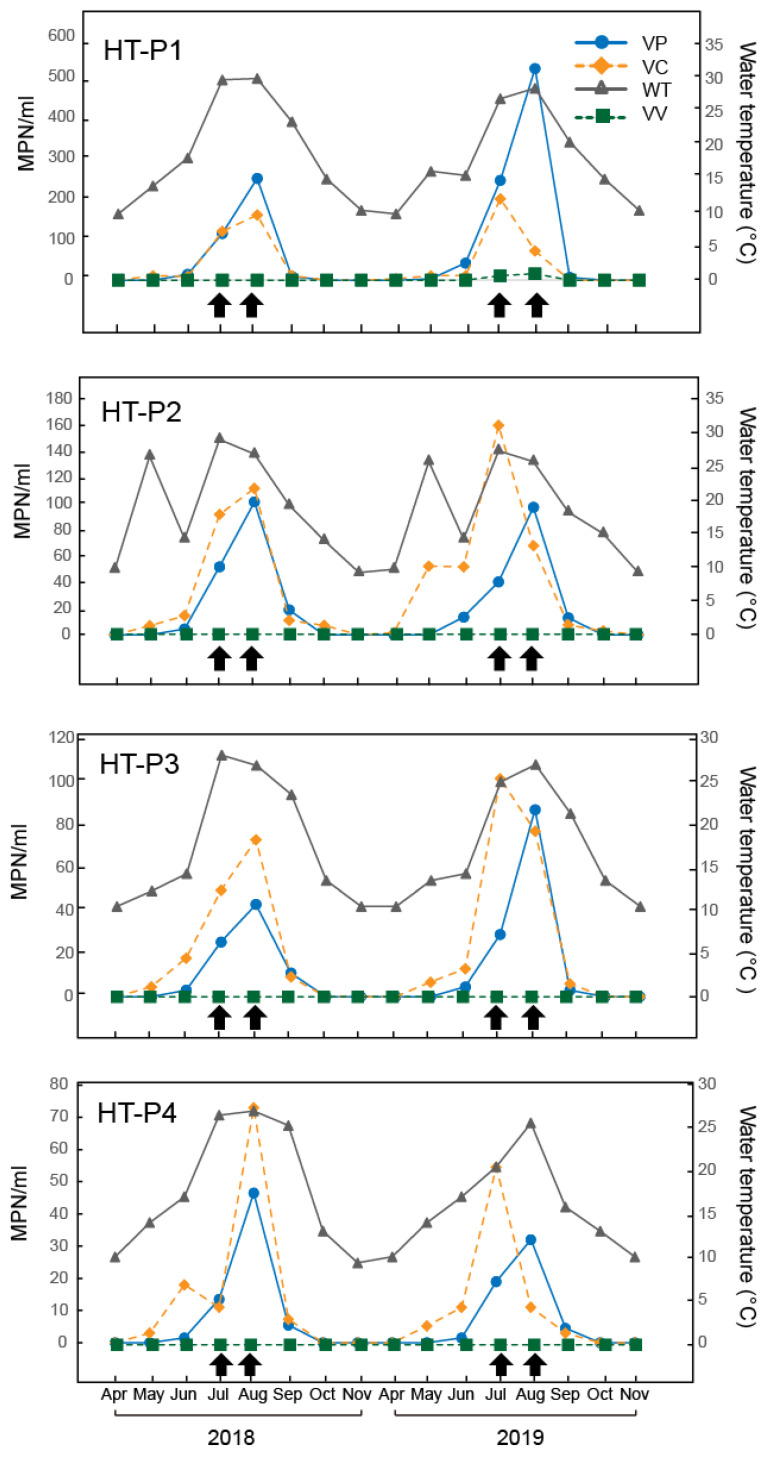
Relationship between the most probable number (MPN) of *V. cholerae* and *V. parahaemolyticus* and water temperature measured at the five sampling sites on the HT River. *V. cholerae* (VC), *V. parahaemolyticus* (VP), *V. vulnificus* (VV) and water temperature (WT). Large black arrows indicate an increase in river depth after heavy rain.

The seasonal and spatial dynamics of *Vibrio* species in the HT River were investigated in the spring, summer, and autumn in 2018 and 2019. Overall, all the locations exhibited higher abundances of *Vibrio* spp. during the summer than during the spring/autumn, and the differences between summer and spring/autumn were statistically significant (*p* < 0.01) (Figure 2). On a spatial scale, significant differences in the abundance of *V. cholerae* and *V. parahaemolyticus* were observed (*p* = 0.018 and *p* = 0.017). Moreover, on a temporal scale, the abundance variability of *V. cholerae* and *V. parahaemolyticus* was also significant (*p* = 0.001 and *p* = 0.02), which indicates that their abundance might be associated with water temperature.

*V. cholerae* was detected simultaneously in five surveyed sites from May to October and varied from below the limit of detection (LOD) to 670 MPN/mL, with a median of 67.7 MPN/mL. From the estuary to HT-P5, the concentration of *V. cholerae* decreased from 195 to 3 MPN/mL. Potential pathogenic *ctxA* + *V. cholerae* was not detected throughout the sampling period. Additionally, positive APW broths were streaked onto TCBS agar, and all *V. cholerae* isolates were confirmed as *V. cholerae* non-O1/non-O139.

*V. parahaemolyticus* was detected only in HT-P1, HT-P2, HT-P3, and HT-P4 from May to September. The mean abundance of *V. parahaemolyticus* was 1.23 × 10^2^ MPN/mL in summer and 10 MPN/mL in autumn, while the average abundance in spring was 11.7 MPN/mL. In addition, the abundance of *V. parahaemolyticus* rapidly decreased from the estuary to the inland region. *V. vulnificus*, whose abundance ranged from 11 to 16 MPN/mL, was only detected in two coastal water samples throughout the summer.

### 3.3. Relationship between Pathogenic Vibrio Species and Environmental Parameters

The correlations between pathogenic *Vibrio* spp. and environmental parameters observed over time varied significantly. Across the entire dataset, significant positive correlations were observed between water temperature and *V. cholerae* (rs = 0.667) and *V. parahaemolyticus* (rs = 0.698) (Table 2).

Moreover, the abundance of *V. parahaemolyticus* was moderately correlated with salinity (r = 0.232, *p* = 0.038). Similarly, *V. cholerae* was not only positively correlated with salinity (r = 0.499, *p* < 0.01) but was also significantly correlated with *V. parahaemolyticus* (r = 0.855, *p* < 0.01), which indicates the coexistence of these two pathogens in the river. As *V. vulnificus* was only detected in two coastal water samples, Spearman rank correlation analysis was not performed.

### 3.4. Detection and MLST of Pathogenic Vibrio *spp*. in the HT River

**Figure 3:**
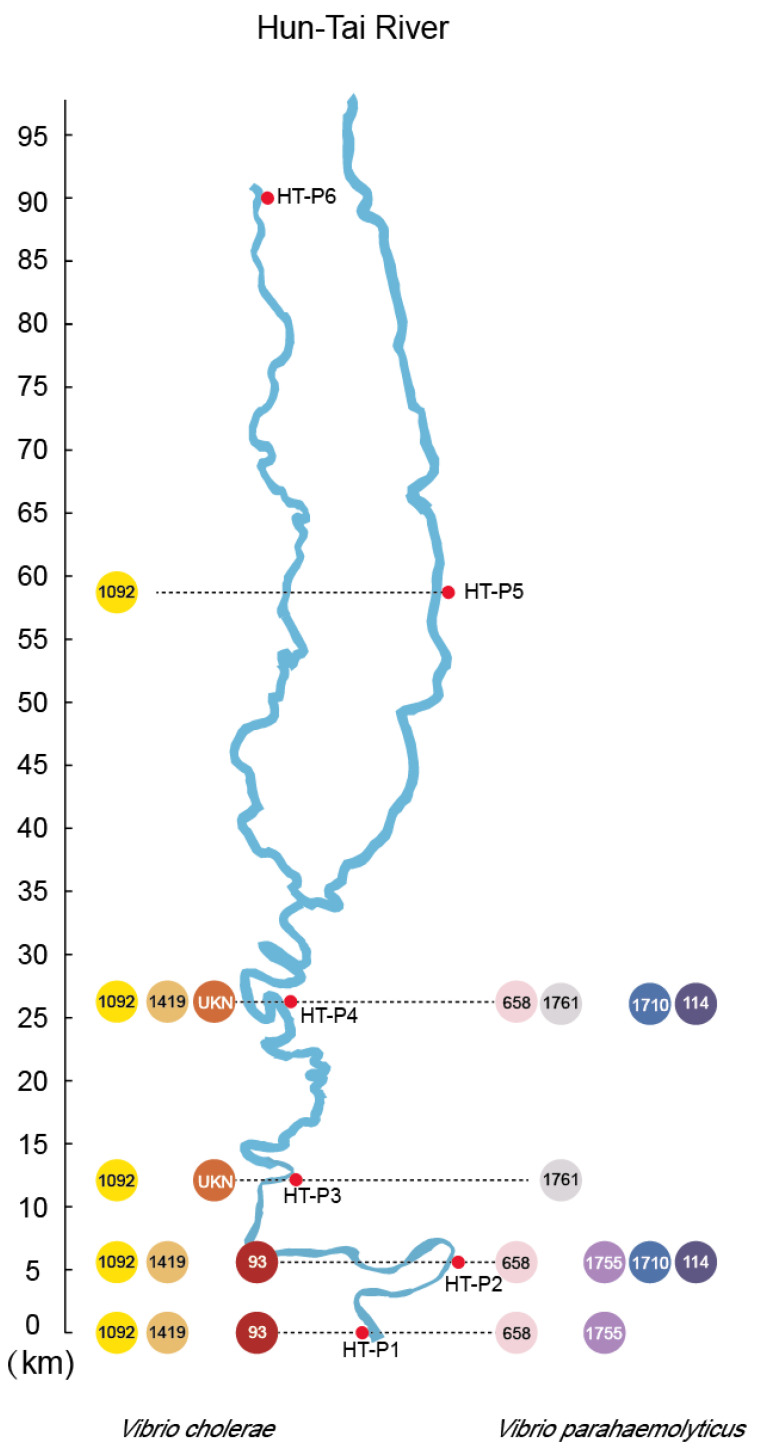
Location of *V. parahaemolyticus-* and *V. cholerae*-positive sampling sites on the HT River. The *V. parahaemolyticus* and *V. cholerae* sequence types in various sampling sites are indicated in the cycles on the left and right side of the rivers, respectively.

In total, 45 strains of *Vibrio* species were isolated, 66.7% of which were *V. parahaemolyticus*. Thirty *V. parahaemolyticus* strains were identified in the HT River (from HT-P1 to HT-P3), whereas 12 *V. cholerae* strains were recovered from all sampling sites (except for HT-P6) (Figure 3). Three *V. vulnificus* strains were only detected in HT-P1.

MLST of *V. parahaemolyticus* and *V. cholerae* was used to subtype the above isolates into 16 and four sequence types (STs), respectively (Tables S2 and S3). One new ST with the absence of the *metE* gene, namely, UKN, was found in *V. cholerae.* MLST of three *V. vulnificus* strains also showed that they belonged to a new ST (Table S4).

Notably, three *V. parahaemolyticus* STs were found in three locations of the HT River (ST658 strains were found in HT-P1, P2, and P3; ST1710 and ST114 strains were both obtained in HT-P2 and P3), which indicates that massive seawater backfilling might have occurred to bring *V. parahaemolyticus* to the inland region.

**Figure 4:**
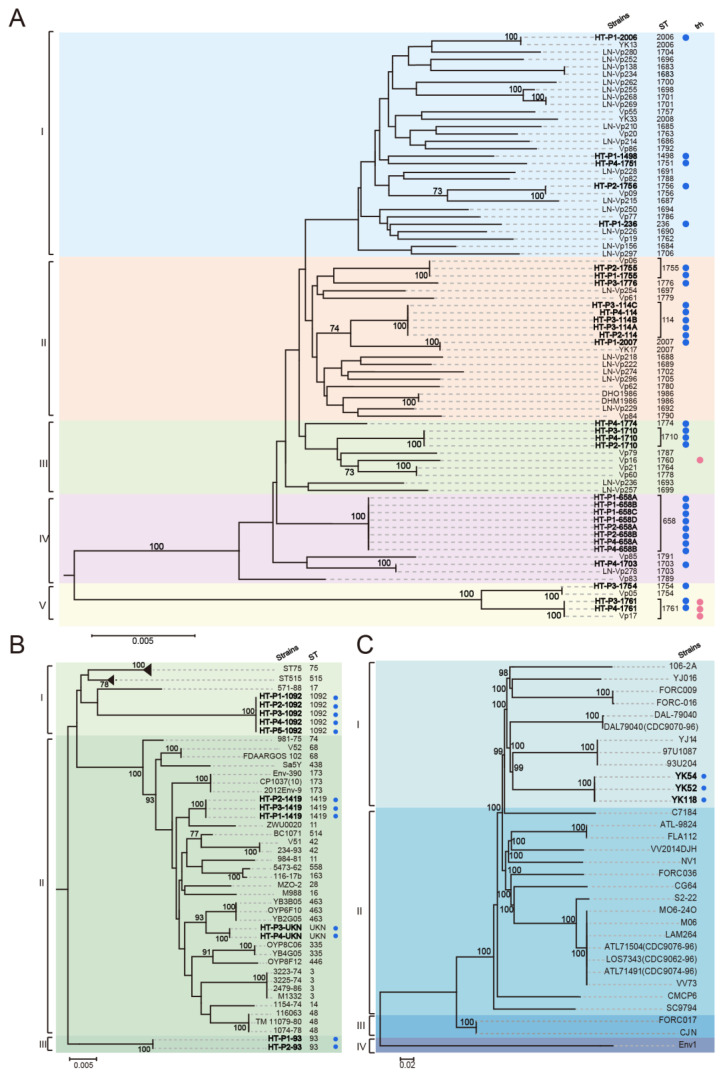
Neighbor-joining tree based on concatenated sequences of seven housekeeping genes from *V. parahaemolyticus* (**A**), *V. cholerae* (**B**), and *V. vulnificus* (**C**). The numbers at the nodes represent bootstrap values based on 1000 replications. The phylogeny was inferred by the neighbor-joining method in MEGA7.0. The strains listed in Tables S2–S4 have been included. UKN: Unknown ST. Blue dot: The strains were isolated in this study. Red dot: *trh* gene was positive.

Together with 50 *V. parahaemolyticus* strains from Liaoning available (pubMLST.org) and 10 isolates collected from Dalian (Table S5), phylogenetic analysis based on concatenated sequences from seven housekeeping genes was conducted. The results showed that 90 strains can be divided into six clusters; *V. parahaemolyticus* strains from the HT River were distributed across five clusters (Figure 4A), of which ST1761, ST1756, and ST2007 were also identified previously in Liaoning. Phylogenetic analysis of 12 non-O1/O139 *V. cholerae* strains with 40 strains from the *V. cholerae* pubMLST database (Table S6) divided them into three clusters (Figure 4B).

We then analyzed the phylogenetic relationships of the isolated *V. vulnificus* strains with the other *V. vulnificus* strains (Table S4). Together with 30 representative public *V. vulnificus* strains, our primary analysis showed that they can be divided into four lineages; three isolated *V. vulnificus* strains were clustered with strain 93U204 and belonged to Lineage I (Figure 4C).

### 3.5. Characterization of Virulence Genes and VSP-I/II Clusters

The PCR results showed that *tlh* was positive for all *V. parahaemolyticus* isolates, but none of the *V. parahaemolyticus* isolates was *tdh* positive. Two ST1761 isolates were *trh* positive. In addition, *vvhA*, *pilA*, *viuB*, and *rtxA* were all detected in three *V. vulnificus* strains. Likewise, *ctxA*, *tcpA*, and *zot* were not detected in any of the *V. cholerae* strains, while *hlyA, rtxA*, and *rtxC* were positive in all isolates.

**Figure 5:**
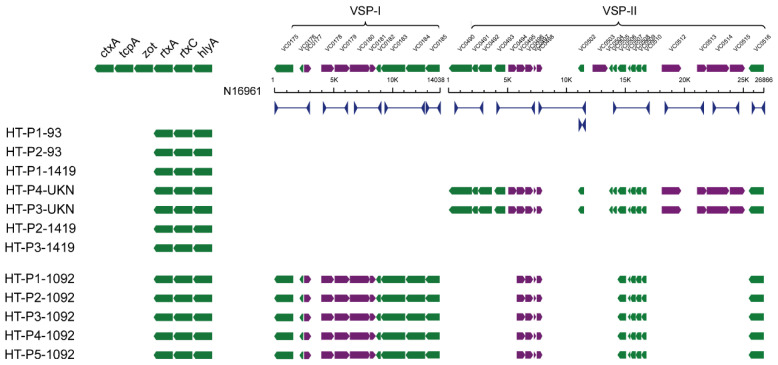
Characterization of virulence genes and *Vibrio* seventh pandemic island I/II (VSP-I/II) clusters in non-O1/O139 *V. cholerae* isolates. PCR primers are shown as blue arrows, and the color gradient from yellow to red indicates the similarity of VSP-I/II clusters in non-O1/O139 *V. cholerae* and strain N16961 (reference).

We performed PCR to detect fiveOpen Reading Frame (ORFs) in the VSP-I cluster and eight ORFs in the VSP-II cluster. The results showed that none of the ST1419 strains and ST93 strains carried VSP-I/II clusters (Figure 5). Strains HT-P3-UKN and HT-P4-UKN harbored VSP-II island genes, but none carried a complete VSP-I island. Five ST1092 strains were positive for both VSP-I and VSP-II clusters. The positive PCR products were further sequenced and compared with those of *V. cholerae* O1 El Tor N16961. For VSP-II in the ST1092 isolates, only 10 ORFs were identified, with 86% (VC0516) to 99% sequence identity with ORFs from *V. cholerae* N16961. These results indicated that the isolates carried incomplete VSP-I/II types.

### 3.6. Antibiotic Resistance Profiles

Antimicrobial susceptibility testing was carried out by using a disk diffusion assay of 10 antibiotics for *V. vulnificus*, *V. parahaemolyticus*, and non-O1/non-O139 *V. cholerae* isolates obtained in this study (n = 45). No significant difference among the sites on the HT River was observed, nor was there a significant difference according to the isolation season. Multidrug-resistant isolates were not detected (Tables S2 and S3). All of the *Vibrio* isolates were sensitive to chloramphenicol, florfenicol, kanamycin, and streptomycin. Of the 45 environmental isolates, 15 isolates showed resistance to two of the antibiotics tested: two showed resistance to both sulfamethoxazole-trimethoprim and penicillin, six *V. cholerae* isolates showed resistance to penicillin and tetracycline, three *V. parahaemolyticus* isolates showed resistance to penicillin and erythromycin (water samples from the HT River) and four exhibited resistance to penicillin and norfloxacin. Intermediate resistance to ciprofloxacin (9.1% of isolates) and norfloxacin (5.4%) was also detected.

## 4. Discussion

### 4.1. Seawater Intrusion Potentially Impacts on the Distribution of Pathogenic Vibrio Species

Diarrhea remains a global public health enigma since contaminated water sources often pose a threat to community health. Although several reports have documented the presence of human pathogenic *Vibrio* spp. in the estuary regions of China, studies regarding the distribution of *V. cholerae*, *V. parahaemolyticus*, and *V. vulnificus* in the inland river regions are scarce. Fukushima and Seki [30] conducted a multi-year study on the ecology of *V. vulnificus* and *V. parahaemolyticus* in brackish environments of the Sada River. In their study, *V. parahaemolyticus* was isolated from river mouths and brackish rivers with an average salinity as low as 4.4 ± 2.0 ppt at cell concentrations of 10^−3^ to 10 MPN mL^−1^. In contrast, similar concentrations of *V. vulnificus* were only isolated from coastal environments with an average salinity 24.0 ± 5.4 ppt, suggesting that both organisms are continuously distributed in the Sada River. In the present study, the combined MPN-PCR method was selected since it has been widely used in studies to assess *Vibrio* species occurrence and dynamics. We have monitored the occurrence and abundance of three major potential human pathogenic *Vibrio* species (*V. cholerae*, *V. parahaemolyticus*, and *V. vulnificus*) in a typical river of north China. The potential pathogenicity and antimicrobial drug resistance of the isolates were also assessed. By monitoring the abundances of pathogenic *Vibrio* species and characterizing the pathogenic species by MLST over 2 years (2018–2019), our 2-year survey revealed that *V. cholerae* were permanently present in all water column fractions during 2018–2019 in the river except for HT-P6. In contrast, the survival of *V. parahaemolyticus* and *V. vulnificus* was only observed in the brackish water area. Notably, five STs of *V. parahaemolyticus* and four STs of *V. cholerae* emerged in the various inland locations of the HT River, indicating that seawater intrusion promoted reversed dissemination of *V. parahaemolyticus* and *V. cholerae* from the estuary to inland.

The association between water temperature and salinity with the occurrence of three pathogenic *Vibrio* spp. has been previously described in different geographic locations, such as in Chesapeake Bay and eastern North Carolina [6,31]. This study confirmed the above observations. The abundance of *V. cholerae* and *V. parahaemolyticus* were both positively correlated with the water temperature and salinity. Interestingly, the abundance of *V. cholerae* was also strongly correlated with the number of *V. parahaemolyticus*, probably because both species are well adapted to the brackish waters [30]. No pathogenic *Vibrio* spp. was identified in April, October, or November, when the water temperature is below 15 °C. In response to decreasing temperature, the three pathogenic *Vibrio* species studied here may enter a viable but non-culturable (VBNC) state in estuarine waters [32]. In early June, when the water temperature rises to 15 °C, both organisms grew in 3 to 10^2^ MPN mL^−1^ of water. The number of both organisms peaked in August when the temperature was highest. Notably, the salinity decreased as the sampling areas moved from the estuary to areas that were further inland. Accordingly, the total abundance of *V. parahaemolyticus* and *V. cholerae* contrasted with the abundance in various sampling sites in the drainage of Wuhan Seafood Market with a higher abundance observed as the estuary was approached. The peaks of *V. parahaemolyticus* and *V. cholerae* at stations HT-P1 and HT-P2 on the HT River coincided with higher salinity values. However, it is still unclear whether the fluctuation of temperature and salinity would induce the VBNC state, which requires future investigation. Overall, this study suggested that seawater intrusion accelerated the transmission of pathogenic *Vibrio* spp. towards the inland areas, which poses a threat to the safety of drinking water.

### 4.2. Identification of Virulence Factors in Pathogenic Vibrio *spp*. and Its Implication for Risk Management of the River

The HT River currently lacks systematic surveillance for potentially human pathogenic environmental *Vibrio* spp. This study provided the first spatiotemporal distribution and pathogenic characteristics of *V. parahaemolyticus* and *V. cholerae* that are present in the river. Several pathogenic *Vibrio* spp. with various virulence factors were identified in inland areas. Interestingly, four STs of *V. parahaemolyticus* were also reported in a previous study that investigated the genetic diversity of *V. parahaemolyticus* in Liaoning Province [33]. This finding suggested that the majority of *V. parahaemolyticus* isolates were likely endemic in this region.

*V. parahaemolyticus* processes various virulence factors, including toxin genes, the type III secretion system (T3SS), thermostable direct hemolysin (TDH), and TDH-related hemolysin (TRH), which are associated with hemolysis and cytotoxicity activity in host cells [34]. The T3SS is an apparatus that secretes and delivers virulence factor proteins directly into eukaryotic host cells [35]. T3SS2 is mainly present in clinical isolates of *V. parahaemolyticus*, while T3SS1 is highly conserved and widespread in both environmental and clinical isolates of *V. parahaemolyticus* [36]. In this study, ST1761 strains isolated from the HT River harbored *trh*, which indicates a potential public health risk posed by these environmental strains. Moreover, the identification of *V. vulnificus*, which was positive for four virulence genes, also signifies a severe threat to the drinking water source and human health.

For *V. cholerae*, three virulence profiles (RTX + *hlyA*, RTX + *hlyA* + VSP-II, and RTX + *hlyA* + VSP-II + VSP-I) were found over the entire study period. This finding is consistent with the observation made by Gong et al. [37], who reported a diverse set of genes among non-O1/O139 strains from the Yangtze River estuary. The cholera toxin (CT) and toxin coregulated pilus (TCP), which are two major virulence factors of the toxigenic *V. cholerae* serotypes O1 and O139, were both absent in two rivers from 2018–2019. However, several accessory virulence-associated genes, including the *hlyA* gene and the RTX cluster, were identified in all of the *V. cholerae* isolates. The *hlyA* gene is a pore-forming cytotoxin that induces vacuolation of eukaryotic host cells and ultimately leads to cell lysis [38]. VSP-II was acquired by the 6th pandemic O1 classical strains via horizontal gene transfer. Taviani et al. (2010) suggested that the presence of this genomic island might confer a selective advantage to toxigenic *V. cholerae* in human hosts [39]. The RTX cluster is commonly present in non-O1/O139 *V. cholerae* strains, and *V. cholerae* strains with RTX toxin alone have caused infections [14]. As these accessory virulence-associated genes have been identified in non-O1/O139 strains from patients with diarrhea, these virulence factors alone or combined might be responsible for the illness. In this setting, precautions should be taken for these environmental strains that pose a potential public health risk.

The fish farms on the HT River were the top three producers of ornamental fish in China. Since August 2017, massive outbreaks of ornamental fish disease occurred in several fish farms on the HT River, resulting in the frequent clean-up of the fish pond and enormous discharges of wastewater [40]. Antibiotics such as florfenicol, fluoroquinolone, sulfamethoxazole, and tetracycline have also been widely used for the treatment of fish diseases in the farms along the river with the frequent emergence of multi-drug-resistant pathogens [40]. Intensive farming forms the bulk of aquaculture and agriculture production along the HT River, which involves high-level inputs of feed as well as high stocking density. The effluent from aquafarms is discharged directly to the rivers through pipelines or open trenches. In addition, due to the extensive use of disinfectants, and the constant threat of oxygen deficiency, nitrification is severely hindered; hence, ammonia and nitrite accumulation takes place.

Therefore, the discharge of the wastewater, combined with the effects of seawater intrusion on the HT River signifies a severe risk to the drinking water source. Adaptive efforts should be focused on the correct treatment of sewage effluent and aquaculture wastewater to avoid the salinization and eutrophication of estuaries and rivers. The development of long-term monitoring of seawater intrusion for drinking and recreational water sources is also urgently needed.

## 5. Conclusions

In conclusion, our study revealed that seawater intrusion was a potential driving force to shape the distribution of pathogenic *Vibrio* spp. in the HT River. We also evaluated the possible pathogenicity of the isolates, suggesting a potential health risk associated with pathogenic *Vibrio* spp. in the HT River. Stringent pollution controls and continuous pathogen surveillance for the water sources in the HT River are suggested to minimize the potential risk of *Vibrio* infection.

## Figures and Tables

**Table 1:** Water temperature and salinity in six sampling sites of the HT River.

Site	Spring		Summer		Autumn	
	WT (°C)	Salinity (‰)	WT (°C)	Salinity (‰)	WT (°C)	Salinity (‰)
HT-P1	13.4 ± 3.3	26.8 ± 1.7	27.8 ± 1.3	24.7 ± 2.4	15.25 ± 5.1	32.3 ± 1.5
HT-P2	13.4 ± 7.6	8.78 ± 1.7	27.5 ± 1.4	4.67 ± 1.6	14.4 ± 4.3	8.08 ± 1.8
HT-P3	12.57 ± 1.8	4.3 ± 0.78	26.8 ± 1.3	1.1 ± 0.8	15.47 ± 5.6	3.1 ± 1.21
HT-P4	13.67 ± 3.1	1.05 ± 0.65	24.9 ± 3.1	0.45 ± 0.2	14.4 ± 5.8	0.41 ± 0.09
HT-P5	13.27 ± 3.2	0.53 ± 0.25	27 ± 1.2	0.12 ± 0.21	13.6 ± 3.1	0.23 ± 0.12
HT-P6	13.1 ± 3.2	0	26.4 ± 3.2	0	13.1 ± 3.2	0

WT: water temperature; salinity was counted at high tide from three samples, then these data were taken from an enumeration of the average salinity.

**Table 2:** Spearman rank correlations (rs) of pathogenic *Vibrio* spp. abundance with environmental variables in the Hun-Tai River.

Variables		*V. cholerae*	*V. parahaemolyticus*
Salinity	rs	0.499 **	0.332 *
	*p*	<0.01	0.038
Temperature	rs	0.667 **	0.698 **
	*p*	<0.01	<0.01
*V. cholerae*	rs	/	0.855 **
	*p*	/	<0.01

* and ** indicate significant differences at *p* < 0.05 and 0.01 levels, respectively.

## Supplementary Materials

The following are available online at https://www.mdpi.com/1660-4601/17/18/6781/s1, Table S1: Primers sequence used for qPCR and PCR amplification. Table S2: Antibiotic resistance profile of *V. parahaemolyticus* strains in HT river. Table S3: Antibiotic resistance profile of *V. chorerae* and *V. vulnificus* strains in HT River. Table S4: *V. vulnificus* isolates used from this study and pubmlst database. Table S5: *V. parahaemolyticus* isolates used from pubmlst database. Table S6: *V. chorerae* isolates used in this study.
