# Supplementary material for: The Impact of Water Intrusion on Pathogenic Vibrio Species to Inland Brackish Waters of China
Source: Int J Environ Res Public Health. 2020 Sep 17;17(18):6781. doi: 10.3390/ijerph17186781 (PMC7558382; doi:10.3390/ijerph17186781)
Supplement: Supplementary file 1 [file ijerph-17-06781-s001.pdf]

International Journal of
Environmental Research and Public Health

MDPI

Table S1. Primers sequence used for qPCR and PCR amplification.

Target Gene	Primer Name	Sequence (5′-3′)	Amplicon Size [bp]	Anneal. Temp [°C]	Reference
16S rRNA	Vibrio-16S	CGGTGAAATGCGTAGAGAT	663	57	Tarr et al., 2007
		TTACTAGCGATTCCGAGTTC			
V. parahaemolyticus					
tdh	tdh-D3F	CCACTACCACTCTCATATGC	425	62	Tada et al., 1992
	tdh-D1R	GGTACTAAATGGCTGACATC			
trh	trh-F R2	GGCTCAAAATGGTTAAGCG	250	62	Tada et al., 1992
	trh-R R6	CATTTCCGCTCTCATATGC			
tlh	tl_884F	ACTCAACACAAGAAGAGATCGACAA	207	60	Noriea et al., 2010
	tl_1091R	GATGAGCGGTTGATGTCCAAA			
toxR	VP toxR 325F	TGTACTGTTGAACGCCTAA	503	55	Neogi et al., 2010
	VP toxR 828R	CACGTTCTCATACGAGTG			
Vibrio vulnificus					
vvhA	vvh-F	TTCCAACTTCAAACCGAACTATGA	205	68	Natividad-Bonifacio et al. 2013
	vvh-R	ATTCCAGTCGATGCGAATACGTTG			
viuB	*viuB*-F	GGTTGGGCACTAAAGGCAGATATA	316	68	Natividad-Bonifacio et al. 2013
	viuB-R	TCGCTTTCTCCGGGGCGG			
pilA	*pilA*-F	TGGCTGCTGTTGCTATTC	217	50	Natividad-Bonifacio et al. 2013
	pilA-R	GGTCCACCACTAGTACCAAC			
rtxA	*rtxA*-F	CGGGATCCTATGGCGTGAACGGCGAAG	1440	61	Natividad-Bonifacio et al. 2013
	rtxA-R	CGGGATCCAGCAGCCACAAGCGATTC			
V. cholerae					
toxR	VC toxR 403F	GAAGCTGCTCATGACATC	275	55	Neogi et al., 2010
	VC toxR 678R	AAGATCAGGGTGGTTATTC			
hlyA	hlyA-744F	GAGCCGGCATTCATCTGAAT	481	60	Rivera et al., 2001
	hlyA-1184R	CTCAGCGGGCTAATACGGTTTA			
zot	zot-225F	TCGCTTAACGATGGCGCGTTTT	947	60	Rivera et al., 2001
	zot-1129R	AAC CCC GTT TCA CTT CTA CCC A			
rtxA	rtxA-F	CTGAATATGAGTGGGTGACTTACG	417	60	Zhou et al., 2016
	rtxA-R	GTGTATTGTTCGATATCCGCTACG			
ctxA	ctxA1	CTCAGACGGGATTTGTTAGGCACG	301	59	Nandi et al., 2000
	ctxA2	TCTATCTCTGTAGCCCCTATTACG			
tcpA	tcpA-F (72F)	CACGATAAGAAAACCGGTCAAGAG	451	60	Rivera et al., 2001
	tcpA-R (477R)	CGAAAGCACCTTCTTTCACGTTG			

VSP-I				
VC0175	VC0175F	TGGATGCTCTCTTCTTCA	2834	O'Shea et al., 2004
	VC0175R	CGCTCACTCACTAATACCGAG		
VC0178	VC0178F	AGAGGCTTGTTTACTATCAG	2053	O'Shea et al., 2004
	VC0178R	ATCGGTACTGTCAGGGCT		
VC0180	VC0180F	GGATGAGCAAATACAGCTAAC	2283	O'Shea et al., 2004
	VC0180R	CTAGGAAGAATTTTATCGGC		
VC0183	VC0183F	CAGTAAGAGTGTAGCGTGCC	3389	O'Shea et al., 2004
	VC0183R	CCTGCACATCGAGATGC		
VC0185	VC0185F	AGGAGGCGTGTAAGTCATAGC	1110	O'Shea et al., 2004
	VC0185R	AGACCACGAATACCTGCTCC		
VSP-II				
VC0490	VC0490F	CGTGAAGGGATATAGGAG	2337	O'Shea et al., 2004
	VC0490R	TGCAGTTGTTGAATGGAC		
VC0493	VC0493F	AATGCTTCTCAGGGGGGTCTT	3600	O'Shea et al., 2004
	VC0493R	CGCTCTTCTTTCCACGCTTCA		
VC0498	VC0498F	AGGTGGTATCGGGCTGGT	4140	O'Shea et al., 2004
	VC0498R	TGCGGCTGGAATGGAGTCTG		
VC0502	VC0502F	TCATCAGTTAGCACACGAAC	476	O'Shea et al., 2004
	VC0502R	GCTATCGTTATACTTGGCG		
VC0504	VC0504F	CAGCAAAGGCGGAAGAGGTAG	3240	O'Shea et al., 2004
	VC0504R	AGCCCGAAATGAATCCCAAAA		
VC0512	VC0512F	CAGTGGCTTCGCAGAGGA	3900	O'Shea et al., 2004
	VC0512R	CCCTCCACTGCTATTCCG		
VC0514	VC0514F	TTATGATCCAAGGAGTAGGG	2089	O'Shea et al., 2004
	VC0514R	AGGCTGAAAAACAACTTGAG		
VC0516	VC0516F	GTTTTCTGCGTTGTTCGAG	965	O'Shea et al., 2004
	VC0516R	TCCTGATGTCTCTCTTGCCG		
VC0517	VC0517F	CCCACTTCTTCCAGAGTATG	1753	O'Shea et al., 2004
	VC0517R	CGCAGTCACAGCTTAAACAAC		

Table S2. Antibiotic resistance profile of *V. parahaemolyticus* strains in HT river.

Site	Strain Name	Date	MLST	Pencillin	Florfenicol	Erythromycin	Chloramphenicol	Ciprofloxacin	Kanamycin	Norfloxacin	Streptomycin	SMZ	Tetracycline
HT-P1	HT-P1-658A	May-18	ST658	R	S	S	S	S	S	S	S	S	S
	HT-P1-658B	Jul-18	ST658	R	S	S	S	S	S	S	S	S	S
	HT-P1-658C	Sep-18	ST658	R	S	S	S	S	S	S	S	S	S
	HT-P1-2006	Aug-18	ST2006	R	S	S	S	S	S	S	S	S	S
	HT-P1-2007	Sep-18	ST2007	R	S	S	S	S	S	S	S	S	S
	HT-P1-658D	Jul-19	ST658	R	S	S	S	S	S	S	S	S	S
	HT-P1-236	Jul-19	ST236	R	S	R	S	S	S	S	S	S	S
	HT-P1-1498	Sep-19	ST1498	R	S	S	S	S	S	S	S	S	S
	HT-P1-1755	Sep-19	ST1755	R	S	S	S	I	S	R	S	S	S
HT-P2	HT-P2-658A	Jul-18	ST658	R	S	S	S	S	S	S	S	S	S
	HT-P2-658B	Jul-18	ST658	R	S	S	S	S	S	S	S	S	S

	HT-P2-114	Sep-18	ST114	R	S	S	S	S	S	S	S	S	S
	HT-P2-1755	Sep-18	ST1755	R	S	R	S	S	S	S	S	S	S
	HT-P2-1756	Jul-19	ST1756	R	S	S	S	S	S	S	S	S	S
	HT-P2-1710	Aug-19	ST1710	R	S	S	S	I	S	R	S	S	S
HT-P3	HT-P3-1761	Jul-18	ST1761	R	S	S	S	S	S	S	S	R	S
	HT-P3-114A	Jul-18	ST114	R	S	S	S	S	S	S	S	S	S
	HT-P3-114B	Sep-18	ST114	R	S	S	S	S	S	S	S	S	S
	HT-P3-1754	Sep-18	ST1754	R	S	S	S	S	S	S	S	S	S
	HT-P3-1710	Jul-19	ST1710	R	S	S	S	S	S	R	S	S	S
	HT-P3-114C	Aug-19	ST114	R	S	S	S	S	S	S	S	S	S
	HT-P3-1776	Sep-19	ST1776	R	S	S	S	S	S	S	S	S	S
HT-P4	HT-P4-658A	Jul-18	ST658	R	S	S	S	S	S	S	S	S	S

HT-P4-658B	Jul-18	ST658	R	S	S	S	S	S	S	S	S	S
HT-P4-114	Sep-18	ST114	R	S	S	S	S	S	S	S	S	S
HT-P4-1761	Jul-19	ST1761	R	S	S	S	S	S	S	S	R	S
HT-P4-1710	Aug-19	ST1710	R	S	S	S	I	S	R	S	S	S
HT-P4-1751	Sep-19	ST1751	R	S	S	S	S	S	S	S	S	S
HT-P4-1774	Sep-19	ST1774	R	S	S	S	S	S	S	S	S	S
HT-P4-1703	Sep-19	ST1703	R	S	S	S	S	S	S	S	S	S

Table S3. Antibiotic resistance profile of V. chorerae and V. vulnificus strains in HT River.

Site	Species	Strain Name	Date	MLST	Pencillin	Florfenicol	Erythromycin	Chloramphenicol	Ciprofloxacin	Kanamycin	Norfloxacin	Streptomycin	SMZ	Tetracycline
HT-P1	V. chorerae	HT-P1-93	Jul-18	93	R	S	S	S	S	S	S	S	S	R
	V. chorerae	HT-P1-1092	Aug-18	1092	R	S	S	S	S	S	S	S	S	S
	V. chorerae	HT-P1-1419	Jul-19	1419	R	S	R	S	S	S	S	S	I	S
	V. vulnificus	YK54	Jul-19	/	R	S	S	S	S	S	S	S	S	S
	V. vulnificus	YK52	Jul-19	/	R	S	S	S	S	S	S	S	S	S
	V. vulnificus	YK118	Aug-19	/	R	S	S	S	S	S	S	S	S	S
HT-P2	V. chorerae	HT-P2-93	Jul-18	93	R	S	S	S	S	S	S	S	S	S
	V. chorerae	HT-P2-1092	Jul-18	1092	R	S	S	S	S	S	S	S	S	R
	V. chorerae	HT-P2-1419	Jul-19	1419	R	S	R	S	S	S	S	S	I	S
HT-P3	V. chorerae	HT-P3-UKN	Jul-19	Unknown	R	S	S	S	S	S	S	S	S	S
	V. chorerae	HT-P3-1419	Aug-19	1419	R	S	R	S	S	S	S	S	I	S
HT-P4	V. chorerae	HT-P4-1092	Aug-18	1092	R	S	S	S	S	S	S	S	S	S
	V. chorerae	HT-P4-UKN	Aug-19	Unknown	R	S	S	S	S	S	S	S	S	S
	V. chorerae	HT-P4-1092	Jul-19	1092	R	S	R	S	S	S	S	S	I	S
HT-P5	V. chorerae	HT-P5-1092	Jul-19	1092	R	S	R	S	S	S	S	S	I	S

Table S4. *V. vulnificus* isolates used from this study and pubmlst database .

Isolate	Country	Year	Source	dtdS	glp	gyrB	lysA	mdh	metG	pntA	purM	pyrC	tnaA	ST (MLST)
YK52	China	2019	River	75	28	1	35	8	25	36	53	77	7	New
YK54	China	2019	River	75	28	1	35	8	25	36	53	77	7	New
YK118	China	2019	River	75	28	1	35	8	25	36	53	77	7	New
DAL 79040 (CDC 9070-96)	USA	1996	Human	37	22	21	29	22	19	1	20	3	13	21
99-796 DP-E7	USA	1999	Shellfish	5	15	16	19	11	7	20	1	4	2	22
99-742 DP-A9	USA	1999	Shellfish	5	15	16	19	11	7	20	1	4	2	22
M06	USA		Human	7	5	3	2	18	14	17	14	22	13	32
LAM264	USA		Human	7	5	3	2	18	14	17	14	22	13	32
ATL 71504 (CDC 9076-96)	USA	1996	Human	7	5	3	2	18	14	17	14	22	13	32
LOS 7343 (CDC 9062-96)	USA	1996	Human	7	5	3	2	18	14	17	14	22	13	32
ATL 71491 (CDC 9074-96)	USA	1996	Shellfish	7	5	3	2	18	14	17	14	22	13	32
ATL-9824	USA	1994	Human	29	21	4	2	24	5	1	15	16	1	36
YJ14	Taiwan	1993	Human	26	28	3	2	3	25	26	21	41	7	76
VV73	Taiwan	1993	Human	7	5	3	2	18	14	17	14	22	13	32
CJN	South Korea	2014	Human	104	5	27	32	23	1	83	1	75	90	294
FDAARGOS_118	USA		Human	5	15	16	19	17	15	2	1	4	2	16
FLA114	USA	1995	Human	5	15	16	19	17	15	2	1	4	2	16
VA-WGS-18028	USA	2007	Environment	5	15	16	19	11	7	20	1	4	2	22
MO6-24/O	South Korea	2011	Human	7	5	3	2	18	14	17	14	22	13	32
93U204	Taiwan	2004	Fish/eel	26	28	3	2	3	25	26	21	41	7	76
97U1087	Taiwan	2008	Fish/eel	26	28	3	2	3	25	26	21	41	7	76
FORC_017	South Korea	2014	Human	104	5	27	32	23	1	83	1	75	90	294
VV2014DJH	RBC	2014	Human	39	5	3	115	32	86	4	21	42	75	442
FORC_036	South Korea		Shellfish	39	59	1	9	3	10	1	4	73	58	460
Env1	USA	2005	Shellfish	166	17	26	162	2	23	5	8	121	119	462
FORC_016	South Korea	2009	Human	167	119	93	163	91	93	51	45	122	102	463
FORC_009	South Korea	2008	Human	167	119	93	163	91	93	51	45	122	102	463
CladeA-yb158	Israel	2005	Fish/eel	37	121	27	2	22	94	1	20	67	13	468
FLA112	USA	1994	Human	29	16	4	2	24	5	1	15	16	53	478
NV1	Taiwan	2011	Environment	171	1	3	119	4	5	102	45	43	7	480
106-2A	USA		Shellfish	173	127	97	166	3	69	103	83	67	7	487
SC9794	South Korea	2011	Environment	29	5	34	32	18	5	88	23	39	7	503
CG64	Taiwan	2011	Environment	118	5	53	2	32	43	10	22	73	7	508
S3-16	USA	2005	Environment	39	5	27	70	58	25	93	30	67	61	509

Table S5. *V. parahaemolyticus* isolates used from pubmlst database.

Isolate	Year	Country	Continent	Source	Region	ST (MLST)
LN-Vp138	2015	China	Asia	environmental	Dalian City, Liaoning	1683
LN-Vp156	2015	China	Asia	environmental	Dalian City, Liaoning	1684
LN-Vp210	2015	China	Asia	environmental	Dalian City, Liaoning	1685
LN-Vp214	2015	China	Asia	environmental	Dalian City, Liaoning	1686
LN-Vp215	2015	China	Asia	environmental	Dalian City, Liaoning	1687
LN-Vp218	2015	China	Asia	environmental	Dalian City, Liaoning	1688
LN-Vp222	2015	China	Asia	environmental	Dalian City, Liaoning	1689
LN-Vp226	2015	China	Asia	environmental	Dalian City, Liaoning	1690
LN-Vp228	2015	China	Asia	environmental	Dalian City, Liaoning	1691
LN-Vp229	2015	China	Asia	environmental	Dalian City, Liaoning	1692
LN-Vp234	2015	China	Asia	environmental	Dalian City, Liaoning	1683
LN-Vp236	2015	China	Asia	environmental	Dalian City, Liaoning	1693
LN-Vp250	2007	China	Asia	environmental	Dandong City, Liaoning	1694
LN-Vp251	2006	China	Asia	environmental	Dandong City, Liaoning	1695
LN-Vp252	2006	China	Asia	environmental	Dandong City, Liaoning	1696
LN-Vp254	2014	China	Asia	environmental	Dandong City, Liaoning	1697
LN-Vp255	2014	China	Asia	environmental	Dandong City, Liaoning	1698
LN-Vp257	2016	China	Asia	environmental	Dandong City, Liaoning	1699
LN-Vp262	2016	China	Asia	environmental	Dalian City, Liaoning	1700
LN-Vp268	2016	China	Asia	environmental	Dalian City, Liaoning	1701
LN-Vp269	2016	China	Asia	environmental	Dalian City, Liaoning	1701
LN-Vp274	2016	China	Asia	environmental	Dalian City, Liaoning	1702
LN-Vp277	2016	China	Asia	environmental	Dalian City, Liaoning	1700
LN-Vp278	2016	China	Asia	environmental	Dalian City, Liaoning	1703
LN-Vp280	2016	China	Asia	environmental	Dalian City, Liaoning	1704
LN-Vp296	2016	China	Asia	environmental	Dalian City, Liaoning	1705
LN-Vp297	2016	China	Asia	environmental	Dalian City, Liaoning	1706
Vp05	2009	China	Asia	environmental	Liaoning	1754
Vp06	2010	China	Asia	environmental	Liaoning	1755
Vp09	2010	China	Asia	environmental	Liaoning	1756
Vp16	2010	China	Asia	environmental	Liaoning	1760
Vp17	2010	China	Asia	environmental	Liaoning	1761
Vp19	2010	China	Asia	environmental	Liaoning	1762
Vp20	2010	China	Asia	environmental	Liaoning	1763
Vp21	2010	China	Asia	environmental	Liaoning	1764
Vp55	2014	China	Asia	environmental	Liaoning	1757

Vp60	2014	China	Asia	environmental	Liaoning	1778
Vp61	2014	China	Asia	environmental	Liaoning	1779
Vp62	2014	China	Asia	environmental	Liaoning	1780
Vp77	2015	China	Asia	environmental	Liaoning	1786
Vp79	2015	China	Asia	environmental	Liaoning	1787
Vp82	2016	China	Asia	environmental	Liaoning	1788
Vp83	2016	China	Asia	environmental	Liaoning	1789
Vp84	2016	China	Asia	environmental	Liaoning	1790
Vp85	2016	China	Asia	environmental	Liaoning	1791
Vp86	2016	China	Asia	environmental	Liaoning	1792
YK13	2017	China	Asia	environmental	Liaoning	2006
YK33	2017	China	Asia	environmental	Liaoning	2008
YK17	2017	China	Asia	environmental	Liaoning	2007

Table S6. *V. chorerae* isolates used in this study.

Strain	BioSample	Location	Serotype	Isolation Source	*adk*	*gyrB*	*mdh*	*metE*	*pntA*	*purM*	*pyrC*	MLST_Type
2012Env-9	SAMN03104885	NA	Inaba	Water	7	57	15	96	2	1	75	173
1154-74	SAMN03248305	NA	O49	Diarrhea	14	14	4	53	22	17	35	14
Env-390	SAMN04168691	Haiti	NA	Environmental	7	57	15	96	2	1	75	173
Sa5Y	SAMN08813238	USA: CA	NA	Water	2	44	11	64	3	8	43	438
Vibrio cholerae str. BC1071	SAMEA104115281	NA	NA	patient sample	14	5	14	43	24	1	149	514
V51	SAMN02435835	NA	NA	NA	25	5	22	16	21	8	11	42
MZO-2	SAMN02435870	NA	NA	NA	2	23	15	42	18	13	10	28
V52	SAMN02435881	Sudan	O37	NA	7	11	4	17	23	1	35	68
CP1037(10)	SAMN00622034	Mexico	NA	Stool	7	57	15	96	2	1	75	173
116063	SAMN00989311	Brazil	NA	Stool	26	5	14	54	28	14	45	48
TM 11079-80	SAMN02393813	NA	NA	NA	26	5	14	54	28	14	45	48
984-81	SAMN02693893	India	O89	Diarrhea	12	5	3	33	31	18	26	11
981-75	SAMN02693890	India	O65	Diarrhea	7	12	14	37	12	1	36	74
571-88	SAMN02693881	China	O105	Diarrhea	14	38	14	43	8	21	11	17
234-93	SAMN02693884	India	O141	Diarrhea	25	5	22	16	21	8	11	42
ZWU0020	SAMN03021537	USA	NA	Intestine	12	5	3	33	31	18	26	11
V. cholerae 116-17b	SAMEA3257723	India	NA	Water	26	5	45	50	31	14	45	163
YB2A06	SAMN03488007	USA	NA	Pond Water	14	81	14	43	99	71	132	335
YB2G01	SAMN03488008	USA	NA	Pond Water	14	81	14	43	99	71	132	335
MZO-2	SAMN05590404	Bangladesh	NA	Human	2	23	15	42	18	13	10	28
3223-74	SAMN05590420	Guam	NA	Storm Drain	1	15	19	15	24	5	14	3

2479-86	SAMN05590416	USA	NA	Moore Swab	1	15	19	15	24	5	14	3
3225-74	SAMN05590421	Guam	NA	Storm Drain	1	15	19	15	24	5	14	3
1074-78	SAMN05590422	Brazil	NA	Sewage	26	5	14	54	28	14	45	48
V52	SAMN05590436	Sudan	NA	Human	7	11	4	17	23	1	35	68
OYP8C06	SAMN07350487	USA	NA	Oyster Pond	14	81	14	43	99	71	132	335
M1332	SAMN08741629	Russia	NA	Feces	1	15	19	15	24	5	14	3
YB4F05	SAMN03488015	USA	NA	Pond Water	14	81	14	43	99	71	132	335
YB2G05	SAMN03488009	USA	NA	Pond Water	18	5	15	48	17	1	45	463
YB3B05	SAMN03488011	USA	NA	Pond Water	18	5	15	48	17	1	45	463
YB4G05	SAMN03488016	USA	NA	Pond Water	14	81	14	43	99	71	132	335
OYP6F10	SAMN07350483	USA	NA	Oyster Pond	18	5	15	48	17	1	45	463
M988	SAMN04376926	Turkmenistan	NA	Water	14	31	20	39	16	1	13	16
HT-P1-1092					18	41	174	48	65	1	4	1092
HT-P2-1092					18	41	174	48	65	1	4	1092
HT-P3-1092					18	41	174	48	65	1	4	1092
HT-P4-1092					18	41	174	48	65	1	4	1092
HT-P5-1092					18	41	174	48	65	1	4	1092
HT-P1-1419					2	5	14	47	6	1	5	1419
HT-P2-1419					2	5	14	47	6	1	5	1419
HT-P3-1419					2	5	14	47	6	1	5	1419
HT-P3-UKN					2	84	14		25	1	227	UKN
HT-P4-UKN					2	84	14		25	1	227	UKN
HT-P1-93					39	27	14	67	50	1	42	93
HT-P2-93					39	27	14	67	50	1	42	93
